# Organic–Inorganic Lead Halide Perovskite Single Crystal: From Synthesis to Applications

**DOI:** 10.3390/nano12234235

**Published:** 2022-11-28

**Authors:** Zhenye Liang, Chen Tian, Xiaoxi Li, Liwei Cheng, Shanglei Feng, Lifeng Yang, Yingguo Yang, Lina Li

**Affiliations:** 1 Zhangjiang Laboratory, Shanghai Synchrotron Radiation Facility (SSRF), Shanghai Institute of Applied Physics & Shanghai Advanced Research Institute, Chinese Academy of Sciences, Shanghai 201204, China; 2 University of Chinese Academy of Sciences, Beijing 100049, China; 3 School of Microelectronics, Fudan University, Shanghai 200433, China

**Keywords:** perovskite single crystal, solar cell, photodetector, light-emitting diode

## Abstract

Organic–inorganic lead halide perovskite is widely used in the photoelectric field due to its excellent photoelectric characteristics. Among them, perovskite single crystals have attracted much attention due to its lower trap density and better carrier transport capacity than their corresponding polycrystalline materials. Owing to these characteristics, perovskite single crystals have been widely used in solar cells, photodetectors, light-emitting diode (LED), and so on, which have greater potential than polycrystals in a series of optoelectronic applications. However, the fabrication of single-crystal devices is limited by size, thickness, and interface problems, which makes the development of single-crystal devices inferior to polycrystalline devices, which also limits their future development. Here, several representative optoelectronic applications of perovskite single crystals are introduced, and some existing problems and challenges are discussed. Finally, we outlook the growth mechanism of single crystals and further the prospects of perovskite single crystals in the further field of microelectronics.

## 1. Introduction

In the past decade, organic–inorganic lead halide perovskites have attracted enormous attention in the optoelectronic field due to their excellent carrier transport ability, great light absorption ability, and long carrier diffusion length. The power conversion efficiency (PCE) of perovskite solar cells (PSCs) based on perovskite polycrystalline films has reached 25.7% [1], which is close to the Shockley–Queisser limitation. However, polycrystalline materials have a large number of grain boundaries and defects, which are easily damaged by water and oxygen in the air, resulting in degradation and affecting the photoelectric performance. Nevertheless, defect passivation and component engineering can effectively improve the efficiency and stability of polycrystalline PSCs [2,3,4,5]. In contrast, a perovskite single crystal has greater potential in photoelectric applications as an absorption layer due to the absence of grain boundaries, high carrier mobility, low trap density, and long carrier diffusion length, which is expected to further improve device performance. The trap density of a MAPbI_3_ single crystal is 4–5 orders of magnitude different from a polycrystalline film [6,7,8]. In recent years, perovskite single crystals have been widely used in PSCs, photodetectors, and LED. Chloride−based perovskite single crystals have a wide bandgap and strong absorption in the ultraviolet range, which is suitable for application in ultraviolet photodetectors [9,10]. Maculan et al. [11] used the inverse temperature crystallization (ITC) method to grow high−quality MAPbCl_3_ single crystals with a suitable optical bandgap, and prepared an efficient visible−blind UV photodetector. Iodine−based perovskite single crystals are mainly used in solar cells and detectors [12,13], while bromine−based perovskite solar cells are mainly used in solar cells, photodetectors, and LED [14,15,16].

However, the thickness of the single crystals will limit the carrier extraction and transmission capacity, and the final device performance and responsiveness are poor. At present, a variety of solution methods have been developed to prepare perovskite single−crystal thin films, which mainly consist of an epitaxial growth method and space−confined methods [17,18]. At present, the growth methods of single crystals are mainly used in ITC [19], the solution temperature lowering method (SLT) [20], the antisolvent vapor diffusion crystallization method (AVC) [21], the solution evaporation method (SE) [22], and so on. However, such factors as a complex preparation process, difficult−to−control film thickness and size, high cost, and difficulty in large area preparation limit their future development. Therefore, how to use a simple growth technology to prepare high−quality ultra−thin perovskite single crystal films is the focus of future research. This paper introduces the recent optoelectronic applications of lead halide perovskite single crystals, and discusses the existing advantages, limitations, and challenges.

## 2. Growth Method of Single Crystals

The growth method of a perovskite single crystal determines its quality, which is one of the most important factors affecting the performance of single crystal devices.

At present, conventional single−crystal growth methods can be summarized as follows:

(1) Inverse−temperature crystallization (ITC) method. This method is used to find a single crystal at low temperature has a greater solubility of the solvent. As the temperature increases, single crystals precipitate and grow because of the reduced solubility.
(2) Antisolvent crystallization (AVC) method. In this method, the organic solvent with low solubility of a single crystal is evaporated into the precursor solution to precipitate single crystals.
(3) Solution−temperature−lowering (SLT) method. This traditional crystallization method reduces the solubility of a single crystal in a solvent by reducing the temperature of solution.
(4) Bridgman growth method. This method is often used for the growth of bulk perovskite single crystals.

Next, we will briefly introduce the growth of organic–inorganic and all−inorganic lead hybrid perovskite single crystals.

**Figure 1:**
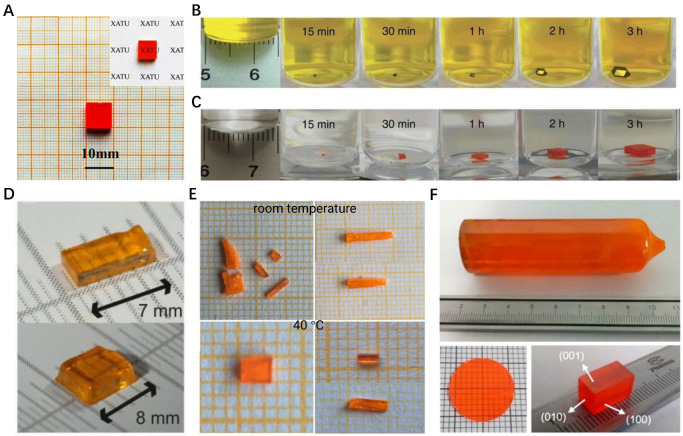
(**A**) Photo of MAPbBr_3_ growing after diluting the antisolvent [23]. Copyright 2019, Springer. (**B**) Growth process of MAPbI_3_ and (**C**) MAPbBr_3_ single crystals from room temperature to 80 °C [25]. Copyright 2015, Nature Publishing Group. (**D**) Photographs of the obtained CsPbBr_3_ SCs in the ITC method [30]. Copyright 2016, American Chemical Society. (**E**) Photographs of CsPbBr_3_ crystals grown at room temperature and 40 °C in the AVC method, respectively [21]. Copyright 2017, American Chemical Society. (**F**) Photographs of the as−grown CsPbBr_3_ crystals in the Bridgman method [31]. Copyright 2018, American Chemical Society.

At present, among organic–inorganic lead halide perovskite single crystals, CH_3_NH_3_PbX_3_ (MAPbX_3_) (X = Cl, Br, I) single crystals are more widely concerned than HC(NH_2_)_2_PbX_3_ (FAPbX_3_) single crystals. Currently, there are SE, ITC, AVC, and SLT for the growth of a MAPbX_3_ single crystal. Figure 1A shows a MAPbBr_3_ single crystal grown by the AVC method. The traditional AVC method can cause the single crystal to grow too fast and reduce the crystal quality. The crystal quality can be improved by adjusting the concentration of a precursor solution and diluting the antisolvent. Shen et al. [23] used dichloromethane (DCM) diluted with N, N−dimethylformamide (DMF) to grow single crystals, resulting in a smoother and more transparent MAPbBr_3_ single crystal with the size increased to 9 × 9 × 3.5 mm^3^. Ruan et al. [24] synthesized the mixed cationic perovskite single crystal FA_x_MA_1−x_PbI_3_ by the AVC method. They used GBL as a solvent to prepare I−based perovskite single crystals. They found that increasing the FA ratio in the Raman spectrum would lead to the blue shift of the peak of the FA bending mode (511 cm^−1^) and the red shift of the peak of the MA mode (958 cm^−1^).

Compared with other solution methods, the ITC method has the advantages of simple operation and low cost. It is worth noting that different halogen−based MAPbX_3_ single crystals require different solvents for growth. For example, DMF is suitable for MAPbBr_3_ as a solvent, while MAPbI_3_ is suitable for growing in γ−butyrolactone (GBL). Bark et al. [25] proposed a fast ITC method to obtain high−quality MAPbX_3_ (X = Br, I) single crystals in a short time. Figure 1B,C shows the growth process of MAPbI_3_ and MAPbBr_3_ single crystals by the ITC method. Topwal et al. [22] grew an 8 × 5 × 1 mm^3^ MAPbCl_3_ single crystal by the SE method. Unlike the solvents used in the ITC method, they dissolved MACl and PbCl_2_ in dimethyl sulfoxide (DMSO) and GBL, resulting in highly transparent colorless single crystals after 2 weeks. Although the SE method and STL method provide a simple and convenient approach for the growth of large−sized single crystals, these two methods have the disadvantage of being time−consuming, usually several weeks. Sun et al. [26] rapidly grew MAPbI_3_ single crystals of 20 × 18 × 6 mm^3^ by the STL method in a chlorine−containing solution within 5 days. The trap density of a single crystal grown by this method is lower than that obtained by the ITC method.

Replacing MA with FA can narrow the bandgap of a perovskite single crystal. Yao et al. [27] combined gradient heating and ITC methods to induce the growth of FAPbBr_3_ single crystals at low temperatures. They dissolved FABr and PbBr_2_ in a mixture of DMF and GBL and grew single crystals at a heating rate of 2–4 °C/day. Bark et al. [28] synthesized 4–5 mm FAPbBr_3_ and α−FAPbI_3_ single crystals by the ITC method. Kundu et al. [29] successfully prepared an α−FAPbI_3_ single crystal using the ITC method, but α−FAPbI_3_ tends to transform into more stable δ−FAPbI_3_ with a larger bandgap at room temperature. Although Bi doping can cause the increase in trap density, it can slow down the δ−phase to α−phase transition time and reduce the transition temperature. The growth of an FAPbCl_3_ single crystal is rarely reported at present.

In addition to the organic–inorganic lead halide perovskite single crystals mentioned above, the growth of all−inorganic lead halide perovskite single crystals has also attracted great attention, among which the growth of CsPbBr_3_ single crystals has been studied more. Kovalenko et al. [30] dissolved CsBr and PbBr_2_ (molar ratio = 1:2) in DMSO and DMSO/cyclohexanol (CyOH)/DMF, respectively. Single crystals nucleated at 90 °C and grew further at 110 °C. Figure 1D shows the photos of CsPbBr_3_ in pure DMSO (7 mm) and DMSO/CyOH/DMF (8 mm). They found that the choice of mixed solvents smoothed the solubility–temperature curve and was more conducive to the growth and repeatability of high−quality single crystals. Ding et al. [21] used methanol as the inverse solvent to grow a CsPbBr_3_ single crystal, as shown in Figure 1E,F, which is a single crystal growing at room temperature and 40 °C, respectively. They found that controlling the evaporation rate of methanol and keeping the temperature at 40 °C contributed to the formation of better bulk single crystals.

The Bridgman growth method involves placing a solution of the precursor of a single crystal in a crucible and then procedurally lowering the temperature so that the single crystal crystallizes in the shape of a grinding tool. By using the Bridgman method, Zhang et al. [31] grew a cylindrical−shaped single crystal with a size of φ24 mm × 90 mm. Then, a 2 mm thick single wafer and a cuboid single crystal were obtained by fine cutting and polishing. He et al. [32] used the Bridgman method to grow superlarge CsPbCl_3_ cylindrical single crystals. The single crystal is more than 3 cm in length and has a thermal conductivity of less than 0.6 W/(m K). At present, the growth of a high−quality CsPbI_3_ single crystal is rarely reported.

In conclusion, in order to quickly obtain high−quality and low−trap−density single crystals, it is necessary to select an appropriate growth strategy and fully consider the factors affecting growth, such as heating/cooling rate, solvent type and ratio, and growth temperature.

## 3. Solar Cells Based on Perovskite Single Crystals

For a solar cell device, there are several reference key performance parameters, such as the short−circuit current density (*J*sc), open−circuit voltage (*V*oc), fill factor (FF), external quantum efficiency (EQE), and power conversion efficiencies (PCEs). Polycrystalline thin−film perovskite cells have developed rapidly in the last decade, and their efficiency has been close to that of monocrystalline silicon cells. However, single−crystal perovskite has received little attention in this field. Polycrystalline films have a large number of grain boundaries and defects, from which water and oxygen can preferentially erode and degrade the films. Although there are many passivation strategies, the single crystal with no grain boundaries and low defects is more potential in the field of cells [33]. In addition, single crystals are an excellent physical platform for studying grain boundaries and ion migration.

**Figure 2:**
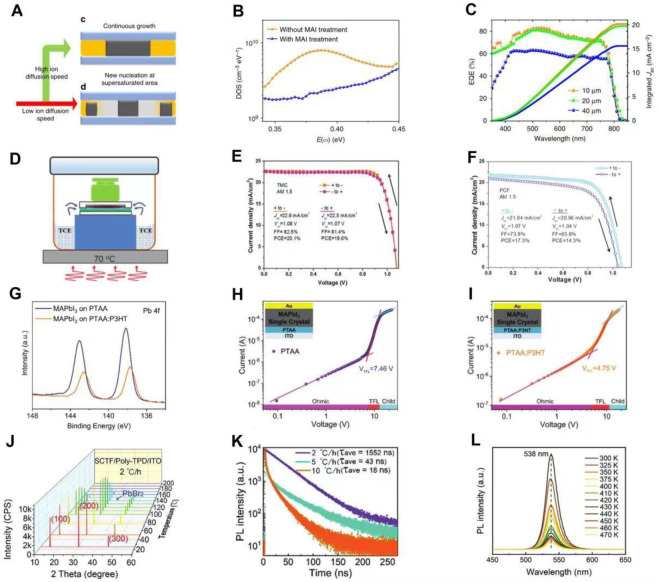
(**A**) The process of growing a single crystal by space restriction. (**B**) Thermal admittance spectroscopy of the single crystals after surface treatment. (**C**) Incident photon−to−electron conversion efficiency (IPCE) and integrated J_SC_ of solar cells with different thickness [34]. Copyright 2017, Springer Nature. (**D**) The process of growing a single crystal by the AVC method. (**E**) Plot of current density versus voltage for a single crystal device. (**F**) Plot of current density versus voltage for a polycrystalline device [35]. Copyright 2020, Wiley. (**G**) The X-ray photoelectron spectroscopy (XPS) of Pb 4*f* for the samples with or without the addition of P3HT. (**H**) J–V curve of the device with PTAA as the hole transporting layer (HTL). (**I**) J–V curve of the device with PTAA:P3HT as the HTL [36]. Copyright 2022, Wiley. (**J**) In situ XRD patterns of MAPbBr_3_ single crystals grown at 2 °C per hour. (**K**) Time−resolved photoluminescence (TRPL) at different heating rates. (**L**) PL spectrum of single crystals from 300 to 470 K [37]. Copyright 2022, Wiley.

The thickness of single crystal perovskite is an important parameter affecting the efficiency of solar cells. If the thickness of a single crystal is too large, the charge recombination will be enhanced, which will eventually lead to the degradation of battery performance. Therefore, the preparation of a suitable thickness of a single−crystal thin film is the focus of attention. Chen et al. [34] proposed an improved method of space−constrained growth of thin single crystals by coating a substrate with poly[bis(4−phenyl) (2,4,6−trimethylphenyl) amine] (PTAA). Such hydrophobic layers can not only constrain the growth of crystals, but also enhance the ion diffusion rate in the substrate gap. As shown in Figure 2A, the growth diagram at different diffusion speeds is shown. PTAA can reduce the formation of small crystals and promote the further growth of single wafers. As shown in Figure 2B, the trap density on the surface of a single crystal can be reduced by two to five times after methylammonium iodide (MAI) treatment. They also further explored the relationship between thickness, optical absorption, and device efficiency. Figure 2C shows the curves of EQE and the integrated current density of an MAPbI_3_ single crystal under different thicknesses. The most appropriate thickness is 10 μm. The excellent cell device of *J*sc is 20.5 mA/cm^2^, *V*oc is 1.06 V, FF is 74.1%, and PCE is 16.1%. Kong et al. [35] grew CH_3_NH_3_PbI_3_ single crystals with a thickness of about 350 nm by combining AVC and spatial confinement. Figure 2D shows the growth device diagram of a single crystal. During the growth process, trichloroethane (TCE) was used as the antisolvent, and then 3−aminopropyl triethoxysilane (APTES) was self−assembled on the surface of silica to form a hydrophobic surface. They controlled the thickness of the single crystal by adjusting the weight of the glass sheet. They also prepared a polycrystalline device for comparison. The structure of the device is Ag/PCBM&BCP/CH_3_NH_3_PbI_3_/ITO/PEDOT:PSS/Glass. The J–V curves of single-crystal devices and polycrystalline devices are shown in the Figure 2E,F. The single−crystal solar cell of *V*oc is 1.08 V, *J*sc is 22.6 mA/cm^2^, the fill factor is 82.5%, and the highest PCE can reach 20.1%. However, the hysteresis effect of polycrystalline devices is significantly larger than that of single-crystal devices, and the PCE in the forward scanning is 17.3%. The small hysteresis in the forward scanning and reverse scanning also indicates the decrease in polarization induced by carrier capture at grain boundaries.

Although the above growth strategy successfully produced a thin single crystal, there is a lattice mismatch between the single crystal and the transport layer, which also leads to a large number of defects at the interface, thus affecting the carrier transport and device performance. Li et al. [36] used hydrophobic poly(3−hexylthiophene) (P3HT) to improve the interface between perovskite and HTL by prompting undercoordinated Pb^2+^ to combine with a sulfur atom. They demonstrated the interaction between P3HT and MAPbI_3_ by X-ray photoelectron spectroscopy and Fourier−transform infrared. Figure 2G shows the comparison sample between HTL with P3HT and pure HTL; a clear peak can be easily found in the core−level diagram of S 2*p*. Besides, in the Pb 4*f* diagram, the intensity of the peak decreased and the peak shifted. The structure of the device is ITO/HTL/MAPbI_3_ perovskite/Au. Figure 2H,I shows the SCLC of the device with and without the treatment of P3HT. The result shows a large reduction in *V*_TFL_, which also represents a reduction in trap density. The optimal cell device has improved PCE from 20.2% to 22.1%. Besides, the *V*_OC_ of the best device is 1.13 V, and *J*_SC_ is 23.88 mA cm^−2^.

Temperature range, heating rate, and precursor solution concentration can affect the quality of the crystal. The study of the nucleation and growth of a single crystal can optimize the growth process of a single crystal. Tang et al. [37] directly grew MAPbBr_3_ single−crystal thin films on the hole transport layer and studied the effects of interfacial energy, heating rate, and precursor concentration between solution and substrate on the quality of a single crystal by in situ XRD. The author set three heating rates of 2, 5, and 10 °C/h, and found that 2 °C/h is the optimal heating rate. The in situ XRD pattern of a single crystal growing at 2 °C/h is shown in Figure 2J. Compared with 5 and 10 °C/h, the PbBr_2_ phase appears at 140 °C for this group of samples, which has higher thermal stability. In addition, the peak strength of the (100) plane is three times and nine times of the other two groups of samples. Figure 2K shows a TPRL diagram of the three groups of samples. It can be seen that the optimal carrier lifetime can reach 1552 ns. Besides, the area–thickness ratio of the single crystal is 1.94 × 10^4^ mm. This also indicates that the slow heating rate is conducive to the generation of high−quality single crystals with low trap density. Figure 2L shows the PL thermal stability test of single crystals grown at a heating rate of 2 °C/h. The emission wavelength is stable between 300 and 470 K, and the intensity decreases with increasing temperature because of the exciton dissociation.

## 4. Photodetectors Based on Perovskite Single Crystals

Photodetectors or X-ray detectors are designed to convert photons into electrical signals. Excellent detectors pursue higher sensitivity, lower detection limit, higher switching ratio, higher EQE, faster response speed, and so on. Common parameters in detector applications are responsiveness (R) and EQE. R reflects the collecting ability of the device to optical signals. R = J_LIG_ − J_DAR_/(P_0_ × S), J_LIG_, and J_DAR_ refer to the current under light and dark conditions, P_0_ is the power density of light, and S is the effective detector area. EQE = Rhc/eλ [38].

**Figure 3:**
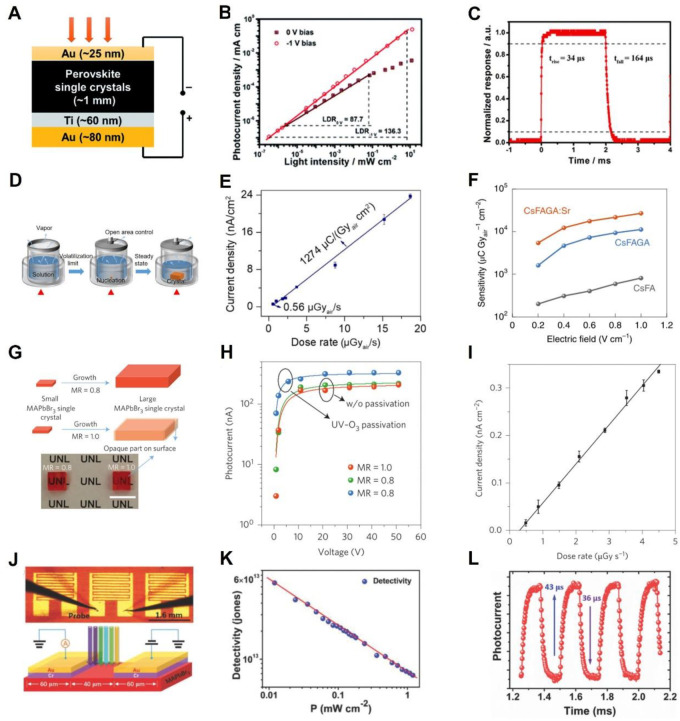
(**A**) Structure diagram of a perovskite single crystal detector. (**B**) LDR of the device at different bias voltages. (**C**) Transient photocurrent response of the single crystal detector [39]. Copyright 2017, Royal Society of Chemistry. (**D**) Schematic diagram of the device for growing a single crystal by changing the open area. (**E**) Changes in the current density of the detector at different dose rates [40]. Copyright 2021, Science Bulletin. (**F**) Plot of sensitivity and electric field for different single−crystal detectors [41]. Copyright 2022, Nature Publishing Group. (**G**) Single−crystal growth images under different molar ratio conditions (MR = PbBr_2_/MABr). (**H**) Device diagram of photocurrent and voltage for different MR and ozone treatment conditions. (**I**) Relationship between current density and dose rate of the device [42]. Copyright 2016, Nature Publishing Group. (**J**) Structure diagram of a single crystal sensor array device. (**K**) Relationship between incident optical power and detection rate of the sensor at a bias voltage of 4 V. (**L**) Transient photocurrent response curve of the sensor [43]. Copyright 2018, Wiley.

MA is a popular cation for the preparation of detectors with lead halogen perovskite. However, the vola−tile nature of MA will reduce the stability of the device. In order to improve the stability, it is necessary to use A−site ions (Cs, FA, etc.) doping to stabilize the perovskite structure. Li et al. [39] synthesized millimeter−size MA_0.45_FA_0.55_PbI_3_ single crystal by ITC method and applied it to the detector, with a structure of Au/perovskite /Ti/Au, as shown in Figure 3A. The trap density of the mixed single crystal is 2.6 × 10^9^ cm^−3^, and the con−ductivity is 1.7 × 10^−7^ Ω^−1^cm^−1^.Compared with FAPbI_3_ and MAPbI_3_, it is improved by about one order of magnitude. The detector has the linear dynamic range (LDR) of 136.3 dB under 870 nm LED light and a bias of −1 V. At 0 V bias, the LDR is 87.7 dB. The detection limit of the detector is lower than 1 nW cm^−2^, as shown in Figure 3B. As shown in Figure 3C, the rise and decay times of the hybrid single−crystal detector in the response time test are 34 μs and 164 μs, respectively. In addition to A−site doping, B−site doping can also im−prove the photoelectric properties of perovskite. Jiang et al. [40] proposed a collaborative strain method to fine−tune the structure of single−crystal perovskite by introducing trace amounts of foreign cations at the A−site and B−site, and thus improve the performance of the detector. This work used the FA−Cs mixed cations, but device performance is low because of the high trap density. They incorporated guanidinium at the A−site to inhibit vacancy formation, and added trace amounts of strontium at B−site to alleviate the microscopic strain between Guanidinium and Pb vacancy after incorporating. Figure 3F shows the relationship between electric field and sensitivity of different doped sample groups. The optimal sensitivity of the device based on doped single crystal perovskite reaches 2.7 × 104 μC Gy_air_^−1^ cm^−2^ at an electric field strength of 1 V cm^−1^, and the lower limit of detection is 7.09 nGy_air_ s^−1^.

A large temperature transition will lead to a large change of crystal growth rate, rapid precipitation of single crystals, and increase in the defects on the surface of bulk single crystals. Therefore, the preparation of high−quality single crystals requires a constant and controllable growth rate method. Li et al. [41] proposed a solvent−volatilization−limited−growth (SVG) strategy to fine−control the growth rate of single crystals, which is universal to hybrid perovskites and inorganic perovskites. Compared with the traditional ITC method, the improved method had higher solute utilization efficiency. Figure 3D shows the design of the single−crystal growth device. A control valve was added to the beaker containing the saturated solution to control the solvent evaporation rate by controlling the exposed area. The trap density of an MAPbBr_3_ single crystal reached 2.8 × 10^8^ cm^−3^. It was an X-ray detector, and the structure of the device was Au/HPSC/PEO/C_60_/bathocuproine/Cr. Figure 3E is a device of the current density and dose rate diagram; the best performance of the device was at 120 kV and a sensitivity of 1274 μC Gy_air_^−1^ cm^−2^. The detection limit was 0.56 μGy_air_ s^−1^. In the AVC method, due to the different solubility of MABr and PbBr_2_ in DMF, the precipitation ratio of MABr and PbBr_2_ is often different from the ideal ratio after antisolvent action, which leads to the reduction of single−crystal quality. Wei et al. [42] reported a sensitive X-ray detector made of an MAPbBr_3_ single crystal. By adjusting the ratio of PbBr_2_ and MABr, they obtained the single crystal with higher transmittance and purer purity. Meanwhile, they found that the single crystal passivated with UV−O_3_ had a lower defect degree and surface recombination speed, as shown in Figure 3G. They compared the single crystal grown with two kinds of precursor solutions (PbBr_2_/MABr = 0.8 and 1.0). It is found that when the ratio is 0.8, the transmittance of the single crystal is higher, and the defects of the bulk phase and surface are lower. Figure 3H shows the photoconductivity test of the single crystal with these two proportions and the sample passivated by UV−O_3_. After passivation, the surface conformity speed of the single crystal reaches 64 cm s^−1^, which is 18 times larger than that before passivation. Its mobility is 1.2 × 10^−2^ cm^2^ V^−1^. Figure 3I shows an X-ray detector device with a ratio of 0.8 single crystal. The structure of the device is Au/MAPbBr_3_ single crystal/C_60_/bathocuproine (BCP)/Ag or Au. The lowest X-ray detection dose was 0.5 μGy_air_ s^−1^, and the sensitivity was 80 μC Gy_air_^−1^ cm^−2^, which was four times higher than the sensitivity of the α−SE X-ray detector.

From the above example, fine control of the growth rate of the AVC method can obtain high−quality single crystals. This approach can also be applied to ITC methods. A small heating step can make nucleation more uniform and reduce surface defects. Lei et al. [43] grew and synthesized high−quality photoelectric sensors. With benefit from the extremely slow heating rate of low−temperature gradient crystallization, the trap density of a MAPbBr_3_ single crystal is only 6.2 ± 2.7 × 10^9^ cm^−3^, and the carrier mobility is 81 ± 5 cm^2^ V^−1^ s^−1^. They tested its imaging capabilities by integrating an array of 56 photoelectric sensors onto a single crystal surface. A structure and schematic diagram of the single−crystal sensor device are shown in Figure 3J. The maximum responsiveness of this photoelectric sensor is 1.6 × 10^4^ mA W^−1^. In addition, the detectivity can reach 6 × 10^13^ Jones, which is 1 or 2 orders of magnitude higher than most InGaAs and silicon sensors on the market, as shown in Figure 3K. The device has a rise time of 43 μm and a decay time of 36 μm under 532 nm shortcut laser, as shown in Figure 3L.

## 5. LED Based on Perovskite Single Crystals

Perovskite single crystal LED devices have less efficiency roll−down due to the reduction of the Auger electron recombination [44]. Different from direct bandgap semiconductors, perovskite is a promising luminescent material in the LED field because of its characteristics of higher photoluminescence quantum yields, tunable colors, and easy preparation [45,46]. Due to the lack of grain boundaries, bulk defects, and ion migration problems of traditional perovskites, single−crystal perovskite devices should have a higher minority carrier lifetime [47]. 

**Figure 4:**
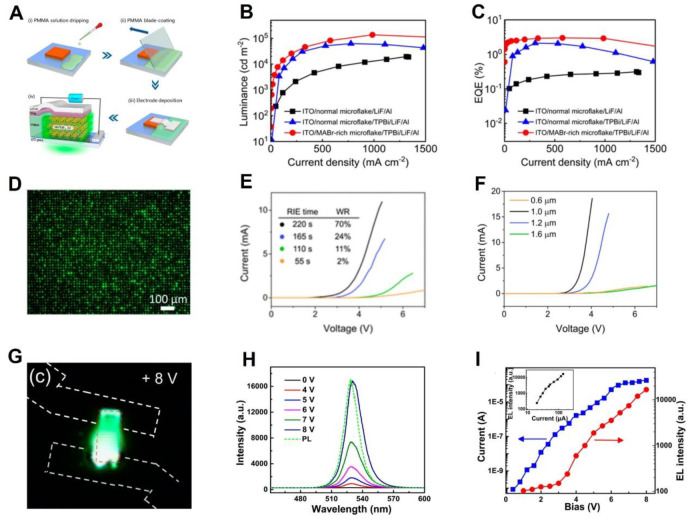
(**A**) The flow of building the device through the LIB method. (**B**) The luminance and (**C**) EQE of the device under different perovskite precursor concentration ratios and structures [44]. Copyright 2022, ACS Publications. (**D**) Surface image of the single−crystal LED after the voltage exceeds the threshold. (**E**) I–V curves of the device at different reactive ion etching times. (**F**) The I–V curves of the devices at different single crystal array thicknesses [48]. Copyright 2020, ACS Publications. (**G**) Color image of an electroluminescence device at a bias of 8 V. (**H**) The EL spectrum at different biases of the device (the PL spectrum is described by a dashed line). (**I**) Plot of EL intensity and current trend at an increased voltage [49]. Copyright 2017, ACS Publications.

Generally, for a bulk single crystal, its thickness is much larger than its diffusion length, which is not conducive to carrier injection. Growing thin single crystals at a micro/nano size can ameliorate this problem. However, the growth of a single crystal is limited by the aspect ratio, and the lateral dimension of a single crystal is not large enough. The prepared device is prone to problems of short−circuit and leakage current. In order to circumvent the problem, Zhang et al. [44] used the method of growth restriction and hydrophobic treatment to grow MAPbBr_3_ single−crystal sheets and then proposed the liquid−insulator bridging (LIB) method to construct LED devices. The detailed process is shown in Figure 4A. The experiment compared the device with 2,2′,2″−(1,3,5−benzinetriyl)−tris(1−phenyl−1−H−benzimidazole) (TPBi) layer and micro−excess MABr. They found that the addition of the TPBi layer facilitates electron transport from the electrode to the perovskite and reduces radiation, and that the micro−excess MABr modulates valence band energy levels. Figure 4B,C show the brightness of the device and EQE curve, the brightness of the best device is 136,100 cd m^−2^, and the best efficiency is 3.0%.

The more popular technology in the semiconductor industry is lithography, but the lithography process requires polar solvents (water, acetone) that will degrade the structure of perovskite. Single−crystal LED applications require further gentle and appropriate device construction methods. Li et al. [48] made a CsPbBr_3_ single−crystal LED device applied to the array type, and the structure of the device is ITO/NiO/perovskite/PMMA/ZnO/Ag. Figure 4D shows a luminescence diagram of the device after etching. They used a column template of polydimethylsiloxane to limit the growth of single crystals. After the template is removed, the polymethyl methacrylate is coated with a spin, and the exposure degree of single crystal is controlled by controlling the time of oxygen reactive ion etching. They optimized the device performance by adjusting the insulating layer and the size of the single crystals. With the increase in reactive ion etching time, the degree of surface exposure of the single−crystal array and its working ratio (WR) both increased. As shown in Figure 4E, when the etching time of the device was 220 s, the WR of the device reached 70%. In addition, they adjusted the height of the single−crystal pixels by changing the concentration of the precursor solution. Figure 4F shows the I–V curves of four groups of devices with different single−crystal heights. These single crystals of different thicknesses all have similar aspect ratios. It can be seen from the figure that the conduction voltage of 0.6 and 1.6 μm devices is nearly twice as large as that of the other two groups. This irregular structure is due to the fact that a single crystal with a smaller height has a smaller surface area due to the aspect ratio, which increases the resistance. When the height is increased, the diffusion length of a few carriers will be exceeded and the mobility will be reduced, so that the conductivity of the device will be increased.

Hu et al. [49] proposed direct crystallization of CsPbBr_3_ on pretreated indium tin oxide substrates by vapor deposition. CsPbBr_3_ has a bridge structure on the overcast etched ITO. When the device is under a bias of 8 V, bright green emission light can be seen, as shown in Figure 4G. When the bias of the device is 6–8 V, a significant increase in EL intensity can be seen, as shown in Figure 4H. From the relationship between voltage and EL intensity in Figure 4I, it can be seen that the EL intensity increases by an order of magnitude from 6 to 8 V.

## 6. Outlook

Large−area fabrication is still a major challenge in the application of single-crystal devices. The growth strategies of bulk single crystals mainly revolve around AVC, ITC, and SE, while the growth strategies of thin single crystals revolve around the combination of a space−limiting method or epitaxial growth method with the former methods. The nucleation and size of crystals have not been accurately and conveniently regulated by these methods. In the future, the growth mechanism of a single crystal or single−crystal thin film can be further studied. For example, in situ XRD can be used to study the dynamic growth process of crystals. In addition, the space−limiting method of thin single crystals still fails to control the thickness and size of the crystal simultaneously, the process of the epitaxial method is complex, and the growth process needs to be further simplified to achieve large−area manufacturing and industrial production [50].

The internal defect density of a single crystal is very small, but the surface and interface defect density are much larger than the internal defect density. These defects can affect the transport ability of carriers from perovskite to the transport layer and the performance of the device. It is hoped that the nonradiative recombination in the device can be reduced by the surface passivation engineering and the interface engineering between the perovskite and the transmission layer.

## Data Availability

Not applicable.
